# Effects of High Temperature on Development, Survival, and Antioxidant Responses of Immature *Monolepta hieroglyphica*

**DOI:** 10.3390/insects17050489

**Published:** 2026-05-11

**Authors:** Rongrong Shi, Jing Lou, Danmei Zhen, Junfeng Kou, Qinglei Wang, Chunqin Liu, Qing Yang

**Affiliations:** 1 Hebei Key Laboratory of Soil Entomology, Cangzhou Academy of Agriculture and Forestry Sciences, Cangzhou 061001, China; shirongrongabc@163.com (R.S.); koujunfeng2021@163.com (J.K.); wqlei02@163.com (Q.W.); 2 Cangzhou Research and Development Center for Biotechnology of Saline-Alkali Land, Department of Agricultural and Animal Husbandry Engineering, Cangzhou Technical College, Cangzhou 061001, China; loujinglj123@163.com; 3 Rural Agriculture Bureau of Jinzhou, Jinzhou 052200, China; zdm920530@126.com

**Keywords:** *Monolepta hieroglyphica*, high temperature, adaptability, developmental duration, antioxidant enzymes

## Abstract

*Monolepta hieroglyphica* Motschulsky (Coleoptera: Chrysomelidae) (*M. hieroglyphica*) is widely distributed in China. Its larvae are soil pests that cause severe damage to the seeds and roots of economically important crops such as corn, cotton, and millet. This study investigated the effects of four temperatures (25, 28, 31, and 34 °C) on the survival rate, food consumption (3rd instar), pupation rate, emergence rate, biometric indices (weight and length), and antioxidant enzyme activity of immature *M. hieroglyphica*. High temperatures (31 °C and 34 °C) adversely affected developmental duration, survival rates, and feeding efficiency. The highest pupation rate, emergence rate, and biometric indices were observed at 28 °C, after which these metrics steadily declined as the temperature increased. Notably, emergence was completely inhibited at 34 °C, resulting in the absence of biometric data. These changes correspond with the temperature-dependent regulation of antioxidant enzyme activities (SOD, CAT, GST, and POD). This study identified the optimal temperature range and critical high-temperature threshold for immature *M. hieroglyphica*, providing key biological parameters for predicting population dynamics and outbreak risks under climate warming, and offering a scientific basis for precise monitoring and temperature-based integrated pest management strategies.

## Simple Summary

*Monolepta hieroglyphica* (Coleoptera: Chrysomelidae) is widely distributed in China; its larvae are soil pests that damage the seeds and roots of corn, cotton, and millet. This study identified 28 °C as the optimal temperature for the growth and development of *M. hieroglyphica* larvae. Temperatures exceeding this range significantly reduced survival rates, daily food consumption (third-instar), pupation rates, and adult emergence rates, as well as significantly decreased the body weight and body length of newly emerged adults. At the same time, exposure to high-temperature environments (34 °C) can cause certain antioxidant enzymes, particularly GST and POD, to lose activity. These findings provide fundamental biological parameters for developing a comprehensive pest management strategy for this species and lay the theoretical foundation for population prediction and the establishment of predictive models.

## 1. Introduction

*Monolepta hieroglyphica* Motschulsky (*M. hieroglyphica*) is classified under the family Chrysomelidae within the order Coleoptera. As an explosive polyphagous insect pest, it infests major crops including maize, soybean, sunflower, and sugar beet in the arid agricultural areas of Northeast, North, and Northwest China [1,2,3]. Adult individuals feed on foliage, pollen, and young silk, whereas larvae reside in the rhizosphere, boring into and feeding on fibrous roots as well as root bark [4,5]. This pest is capable of drastically reducing the photosynthetic area of host plants and elevating their lodging rate, leading to a yield loss ranging from 5% to 15% in ordinary years and exceeding 30% during outbreak episodes [6,7,8]. Over the past 20 years, the combined effects of global warming and agricultural practices—such as conservation tillage, straw incorporation, and high-density planting to enhance yield—have caused a northward shift in the northern distribution limit of the pest (Hulun Buir Plant Protection and Plant Quarantine Center, Release Date: 25 July 2025). The annual number of generations has risen from the conventional 1–2 to 2–3, and the period of infestation has prolonged from the former 30 days to over 60 days. It exhibits an outbreak-prone pattern featuring “earlier onset, increased population peaks, and expanded distribution scope” [9,10,11], and has emerged as a key biotic constraint hampering the green, high-quality, and efficient production of dry grain crops in northern China [1,12,13].

Temperature is the primary abiotic factor influencing insect growth, development, survival, reproduction, and population dynamics [14]. For those pests that move slowly or are embedded in the soil, their mobility and behavioral flexibility during the immature stages are relatively limited. The temperature tolerance range of different insect larvae are different. Studies have shown that the larvae of *Hypothenemus hampei* can develop normally within a temperature range of 15–30 °C [15], while the optimal temperature range for the larvae of *Serangium japonicum* is 20–32 °C [16]. Furthermore, high temperatures have a significant impact on various biological parameters of insects, including pupation rate, eclosion rate [17], as well as adult body weight and body length [18]. Nevertheless, existing studies on *M*. *hieroglyphica* have mainly focused on adult feeding habits, phototactic behavior and chemical control measures [19,20]. Key biological parameters, including the upper thermal survival limits of larvae and the quantitative relationship between pupal developmental rate and temperature, are still poorly understood [21]. Therefore, current regional forecasting models still rely on coarse estimates based on the effective accumulated temperature method from the 1980s, with errors as high as 7–10 days, failing to meet the demands of precise pest control [9].

In recent years, to address the limitations of traditional growth rate temperature models, researchers have begun incorporating antioxidant enzyme systems as immediate response indicators to temperature stress [22]: Superoxide dismutase (SOD) rapidly converts superoxide anion radicals generated by stress into H_2_O_2_, while catalase (CAT) and peroxidase (POD) further decompose H_2_O_2_ into H_2_O and O_2_. These three enzymes synergistically maintain the dynamic equilibrium of reactive oxygen species (ROS) within cells [23]. Their activity levels show a significant positive correlation with insect tolerance to high or low temperature stress [24]. Therefore, measuring the dynamic changes in antioxidant enzyme activity under different temperature stresses can assess the intensity of insects’ immediate responses to extreme temperatures, providing key physiological parameters for revealing differences in their thermal adaptability and predicting potential geographic expansions [25,26].

This study employed a constant temperature (25, 28, 31, 34 °C) as the experimental platform to systematically measure the survival rate, food consumption (3rd-instar), pupation rate, emergence rate, body weight and length, and antioxidant capacity of immature *M. hieroglyphica*. The aim was to evaluate the effects of temperature on underlying the adaptability and heat tolerance of its immature under varying temperatures. This research offers theoretical support for disaster early warning systems and green pest control strategies for *M. hieroglyphica* under changing climate conditions.

## 2. Materials and Methods

### 2.1. Insect Sources

The species *M. hieroglyphica* were collected from pesticide-free corn fields at Xinzhou (Shanxi Province, 38.26° N, 112.39° E) in 2023. Key characteristics used to identify *M. hieroglyphica* included, for example, the presence of a nearly circular light spot at the base of each elytron [27,28]. Next, the field-caught individuals were transferred to the Institute of Plant Protection of the Cangzhou Academy of Agricultural and Forestry Sciences (CAAS; 38.16° N, 116.48° E) in Cangzhou, Hebei Province. The *M. hieroglyphica* species were kept under laboratory conditions. The *M. hieroglyphica* were reared on *Zea mays* L. (corn: five-leaf stage of variety Longsheng, Jinzhong Longsheng Seed Co., Ltd., Xinzhou, China) in screened cages (30 cm × 30 cm × 30 cm) within a controlled climate chamber (RXZ500D, Ningbo Jiangnan Instrument Factory, Ningbo, China) and held at 25 ± 1 °C, 70 ± 5% RH, and 16:8 h (light/dark) photoperiod. Each rearing cage contained 1–2 egg trays for insect egg-laying. Corn leaves were replaced daily, with insect eggs collected regularly for subsequent research after hatching.

### 2.2. Temperature Treatments

All subsequent experiments were conducted in laboratory climate chambers, each monitored using HOBO (temperature loggers, Onset Computer Corporation, Bourne, MA, USA) devices to ensure a constant temperature. Based on the results of the preliminary experiment, four temperatures (25, 28, 31, and 34 °C) were selected. These temperatures represent typical conditions during the growing seasons of corn and other crops in northern China, considering the effects of the greenhouse effect.

### 2.3. Developmental Duration and Survival

Newly hatched (<12 h) *M. hieroglyphica* larvae were removed from the breeding population and placed in Petri dishes (5 cm diameter, 1 cm height). The bottom of each dish was covered with 1% hydrogel, followed by a layer of filter paper that completely covered the agar medium. Corn roots were spread flat on the filter paper to allow free feeding by the larvae, and the medium was replaced every 24 h. One larva was placed per dish, and 150 larvae were randomly selected per group. These leaf beetle larvae were placed in climate chambers at different temperatures (25, 28, 31, or 34 °C, 70 ± 5% RH, and 16:8 h (L/D) photoperiod), respectively. Survival and molting of the larvae were recorded daily, and observations continued until all larvae either reached the pupal stage or died.

### 2.4. Feeding Capacity

As described by Kaufmann [29], we assessed the feeding capacity of third instar larvae of *M. hieroglyphica* (entered the peak feeding period) at the four experimental temperatures and above climatic conditions (70 ± 5% RH, 16:8 (L/D) photoperiod). Third-instar larvae of *M. hieroglyphica* (<12 h of age) that have just shed their skin were starved for 24 h. Next, one larva was transferred to a Petri dish (diameter 5 cm, height 1 cm) containing a piece of corn root (pre-weighed). The Petri dishes without leaf beetle larvae were used as a control. At each experimental temperature, a total of thirty leaf beetle larvae (i.e., replicates) were individually exposed to a given quantity of food items for 24 h. Next, we recorded the quantity of corn root consumed by each individual.

We followed the below formula [29]:

$$Correct\;feeding\;amount=W-\left[L+\frac{aW+bL}{2}\right]$$

where *W* represents the initial mass of the experimental diet at the start of the trial, and *L* stands for the mass of the leftover diet upon completion of the test. The coefficients are calculated as follows:

*a* = (initial mass of the control diet−final mass of the control diet)/initial mass of the control diet;

*b* = (initial mass of the control diet−final mass of the control diet)/final mass of the control diet [29].

### 2.5. Pupa and Eclosion

A layer of moist soil (0.5 cm thick, with soil moisture ranging from 18% to 20% and pH level between 6.5 and 7.0) was laid at the bottom of the Petri dish to provide the pupation conditions for the late third-instar larvae (characterized by ceased feeding and darkened body coloration). The number of pupae was recorded, and the development status of the pupae was observed daily. After the emergence was completed, the number of emerged adults was recorded, and the pupation rate (pupation rate = number of pupae/number of third instar larvae × 100%) and the emergence rate (emergence rate = number of emerged adults/total number of pupae × 100%) were calculated.

### 2.6. Weight and Body Length

Carbon dioxide was used to anesthetize the newly emerged adults (unfed) at each experimental temperature. Subsequently, the weight and body length of each individual adult were measured. For each temperature treatment, 15 pairs of adults (half male and half female) were tested. Insect weight was determined using an electronic analytical balance with a precision of 0.1 mg, while body length was measured with a vernier caliper.

### 2.7. Antioxidant Responses

The larvae of *M. hieroglyphica* at various instars (1st, 2nd, or 3rd instar and <12 h) were placed in culture dishes (diameter 5 cm, height 1 cm) with a bottom layer of 1% agar. Sufficient food was provided, and the larvae were maintained at experimental temperatures of 25, 28, 31, and 34 °C for 120 h. Subsequently, healthy surviving larvae were selected, rapidly immersed in liquid nitrogen, and stored at −80 °C until subsequent laboratory testing. Frozen individuals were then placed in phosphate-buffered saline (PBS, pH 7.4) at a ratio of 1 mL PBS per 0.1 g tissue. Samples were placed in a cold mortar, ground with liquid nitrogen, and subjected to a crude extraction. The mixture was then centrifuged at 4 °C and 10,000× *g* for 10 min. The supernatant was subsequently centrifuged under the same conditions for antioxidant capacity determination. Each treatment had three replicate groups, with 0.1 g of larvae pooled per replicate.

The activity levels of four antioxidant enzymes (i.e., SOD, CAT, GST, and POD) were determined using commercial assay kits (Jianglaibio Co., Ltd., Shanghai, China) following the manufacturer’s instructions. Absorbance was recorded using a light-absorbing enzyme marker (BioTek 800 ™ TS, BioTek Co., Ltd., Winooski, VT, USA), with the activities of SOD, CAT, GST, and POD being detected at 450 nm.

### 2.8. Data Analysis

A one-way analysis of variance was used to analyze the effects of temperature on the larvae longevity, survival, and antioxidants of leaf beetles at various instars. All data were first checked for normality and homogeneity of variance and were transformed when they did not fit a normal distribution. Meanwhile, Tukey’s test was used to determine the differences between different temperatures for the leaf beetle (*p* < 0.05). Survival curves across different temperatures were analyzed by the Kaplan–Meier logrank test. All statistical analyses were conducted using SPSS 25.0 software and Microsoft Excel 2010, while the charts were generated using SigmaPlot 12.5 and OriginPro 9.0.

## 3. Results

### 3.1. Developmental Duration and Survival

Different temperatures significantly affected the developmental duration and mortality of *M. hieroglyphica* larvae (Table 1). The results showed that as the temperature increased, the developmental duration of larvae at each instar generally decreased. However, the duration of the 3rd-instar larvae was significantly prolonged at 34 °C (Tukey’s test: *F*_3,8_ = 10.99, *p* < 0.001). At 28 °C, the mortality rate of larvae at all instars reached its minimum values, whereas at 34 °C, the mortality rate peaked across all instars, with the highest mortality observed in 3rd-instar larvae (66.36 ± 6.36%) (Tukey’s test: first, *F*_3,8_ = 352.79, *p* < 0.001; second, *F*_3,8_ = 25.92, *p* < 0.001; third, *F*_3,8_ = 92.10, *p* < 0.001). Larvae at each instar exhibited the shorter developmental duration and the lowest mortality at 28 °C, indicating that 28 °C is the optimal temperature for the larval stage of *M. hieroglyphica*.

**Figure 1:**
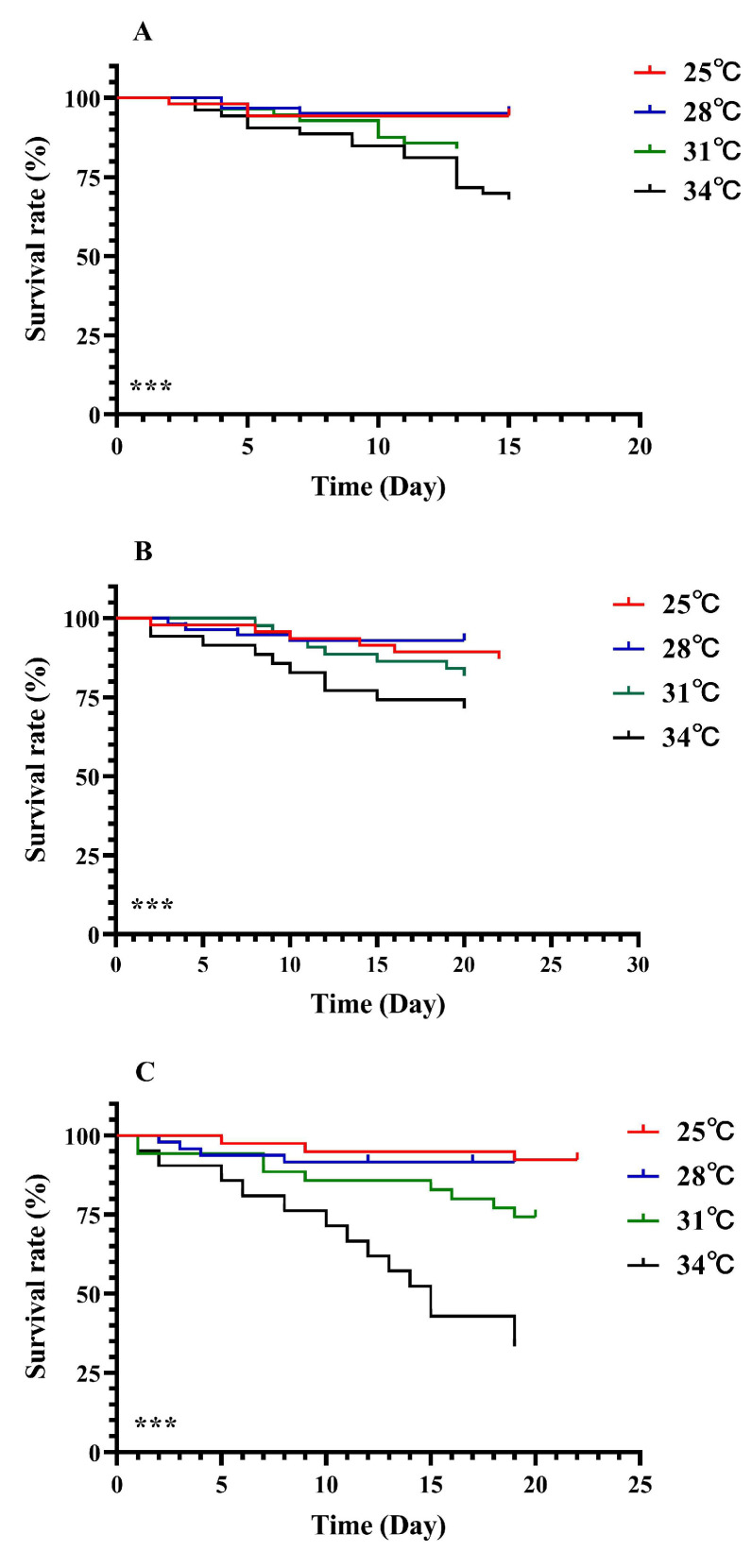
Survival curves of *M. hieroglyphica* larvae at different temperatures during different stages of development ((**A**): First instar larva; (**B**): Second instar larva; (**C**): Third instar larva). Survival statistics were calculated using the Kaplan–Meier survival curve and compared using the logrank test (individuals = 150, ***, *p* < 0.001).

The survival of *M. hieroglyphica* larvae was affected by the temperature (log-rank test: A: *χ*^2^ = 21.05, *df* = 3, *p* < 0.001; B: *χ*^2^ = 148.14, *df* = 3, *p* < 0.001; C: *χ*^2^ = 66.21, *df* = 3, *p* < 0.001; Figure 1). With the increase in temperature (25 °C to 28 °C to 31 °C to 34 °C), the survival rate of the *M. hieroglyphica* larvae decreased significantly. At 34 °C, the survival rate of all larval stages of the *M. hieroglyphica* showed the sharpest decline. On the fifth day of treatment, there was a significant difference in the survival rate of the *M. hieroglyphica* larvae compared to those at other temperatures (Tukey test: A: *F*_3,8_ = 439.73, *p* < 0.001; B: *F*_3,8_ = 171.40, *p* < 0.001; C: *F*_3,8_ = 160.53, *p* < 0.001; Figure 1).

### 3.2. Feeding Capacity

**Figure 2:**
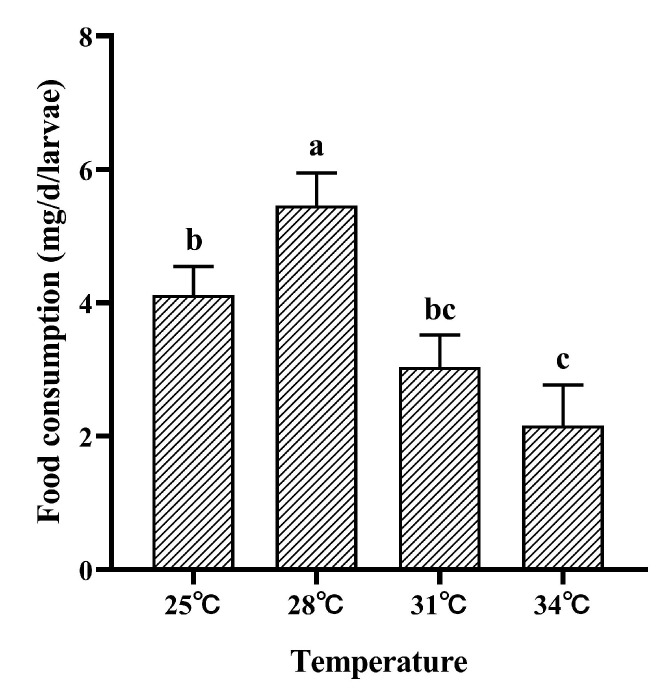
Daily food consumption of third instar larvae of *M. hieroglyphic* at different temperatures. The results are represented as mean ± SE. Different letters above the bars indicate statistically significant differences among temperatures (ANOVA: Tukey’s post hoc test, *p* < 0.05).

Due to their small body size and relatively low damage potential, first- and second-instar larvae of *M*. *hieroglyphica* were excluded from this study. We assessed only the effects of different temperatures on the daily food consumption of third-instar larvae. Our results demonstrated that temperature have significantly influenced the daily food intake of third-instar larvae of *M. hieroglyphica* (Tukey’s test: *F*_3,8_ = 25.56, *p* < 0.001, Figure 2). Specifically, larval food intake increased significantly with rising temperature from 25 °C to 28 °C, peaking at 28 °C (5.46 mg larva^−1^ day^−1^). In contrast, further temperature elevation to 34 °C resulted in a pronounced decrease in food intake, which fell to the minimum value (2.16 mg larva^−1^ day^−1^).

### 3.3. Pupa and Eclosion

**Figure 3:**
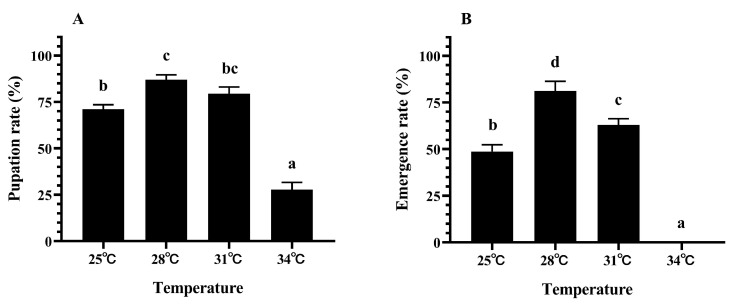
Pupation rate (**A**) and emergence rate (**B**) of *M. hieroglyphica* at different temperatures. The results are represented as mean ± SE. Different letters above the bars indicate statistically significant differences among temperatures (ANOVA: Tukey’s post hoc test, *p* < 0.05).

The pupation and emergence rates of *M. hieroglyphica* are presented in Figure 3 (Tukey’s test: A: *F*_3,8_ = 126.87, *p* < 0.001; B: *F*_3,8_ = 149.89, *p* < 0.001; Figure 3). At 28 °C, *M. hieroglyphica* exhibited optimal pupation and emergence rates of 87.04% and 81.25%, respectively. When temperature increased to 31 °C, both pupation and emergence rates significantly decreased. The negative impact became pronounced at 34 °C, where pupation rate dropped by 68.08 percentage points and adult emergence failed completely (A: *t* = 21.65, *df* = 4, *p* < 0.001; B: *t* = 27.59, *df* = 4, *p* < 0.001; Figure 3).

### 3.4. Weight and Body Length

**Figure 4:**
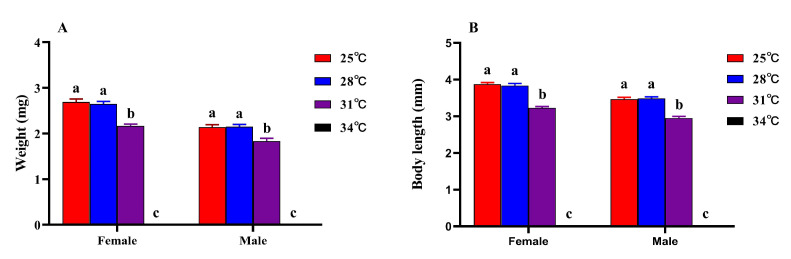
Weight (**A**) and body length (**B**) of *M. hieroglyphica* at different temperatures. The results are represented as mean ± SE. Different letters above the bars indicate statistically significant differences among temperatures (ANOVA: Tukey’s post hoc test, *p* < 0.05).

Temperature stress has a significant negative impact on the body weight of *M. hieroglyphica* adults (newly, <12 h; Tukey’s test: Female: *F*_3,8_ = 1927.71, *p* < 0.001; Male: *F*_3,8_ = 1482.51, *p* < 0.001; Figure 4A; Supplemental Figure S1). At 25 °C and 28 °C, female body weight was maintained at approximately 2.69 mg and 2.65 mg, respectively, while at 31 °C it decreased to 2.17 mg. For males, body weight at 25 °C and 28 °C was 2.14 mg and 2.15 mg, respectively, and declined to 1.83 mg at 31 °C. No data were available for the 34 °C treatment group, indicating potential lethality or developmental arrest at this temperature. At all tested temperatures (except 34 °C), females consistently exhibited higher body weight than males, and both sexes showed a gradual decline in body weight as the temperature increased from 25 °C to 31 °C.

Consistent with the trend of weight changes, temperature also had a negative impact on body length (Tukey’s test: Female: *F*_3,8_ = 6020.29, *p* < 0.001; Male: *F*_3,8_ = 5073.62, *p* < 0.001; Figure 4B; Supplemental Figure S1). In females, body length was 3.87 mm at 25 °C and 3.84 mm at 28 °C, but dropped to 3.23 mm at 31 °C. In males, body length was 3.47 mm at 25 °C and 3.49 mm at 28 °C, and decreased to 2.95 mm at 31 °C. Consistent with survival and weight data, no individuals survived at 34 °C. At all tested temperatures (except 34 °C), females exhibited greater body length than male individuals. Furthermore, within the temperature range of 25 to 31 °C, the body length of both sexes exhibited a negative correlation with increasing temperature.

### 3.5. Antioxidant Responses

**Figure 5:**
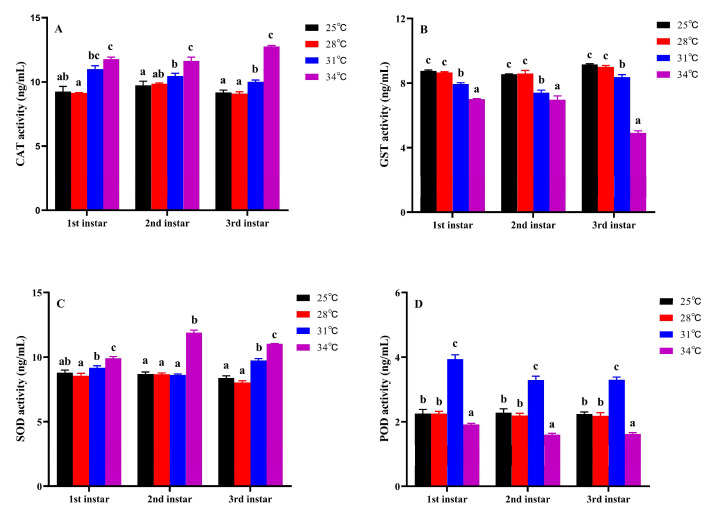
Effects of temperature stress on the detoxification enzyme activity levels of larvae *M. hieroglyphica*. (**A**): CAT activity; (**B**): GST activity; (**C**): SOD activity; (**D**): POD activity. The results are represented as mean ± SE. Different letters above the bars indicate statistically significant differences at *p* < 0.05 (ANOVA followed by Tukey’s post hoc test).

Temperature stress has a significant impact on the antioxidant enzyme activities of *M. hieroglyphica* larvae (Figure 5; Supplemental Table S1). Within the range of 25–28 °C, the activities of CAT, SOD, GST and POD remained relatively stable; as the temperature rose further, CAT and SOD activities showed a continuous upward trend (Tukey’s test: CAT: 1st instar: *F*_3,8_ = 77.50, *p* < 0.001; 2nd instar: *F*_3,8_ = 37.99, *p* < 0.001; 3rd instar: *F*_3,8_ = 472.36, *p* < 0.001; SOD: 1st instar: *F*_3,8_ = 37.31, *p* < 0.001; 2nd instar: *F*_3,8_ = 479.51, *p* < 0.001; 3rd instar: *F*_3,8_ = 302.01, *p* < 0.001; Figure 5A,C), while the activity of GST continues to decline (Tukey’s test: 1st instar: *F*_3,8_ = 696.28, *p* < 0.001; 2nd instar: *F*_3,8_ = 70.77, *p* < 0.001; 3rd instar: *F*_3,8_ = 897.64, *p* < 0.001; Figure 5B); the activity of POD first increases and then decreases, reaching its peak at 31 °C and dropping slightly at 34 °C (Tukey’s test: 1st instar: *F*_3,8_ = 249.73, *p* < 0.001; 2nd instar: *F*_3,8_ = 167.28, *p* < 0.001; 3rd instar: *F*_3,8_ = 273.24, *p* < 0.001; Figure 5D).

## 4. Discussion

Temperature plays a critical role in shaping the development and survival of insect larvae. This study provides new evidence on the impact of high temperatures on the ecological indicators of the larvae *M. hieroglyphica*. In the temperature range of 25–28 °C examined in this study, larval development time decreased as temperature increased, in line with the common trend of faster insect development at higher temperatures. High cumulative survival rates were observed, indicating that this temperature range is ideal for promoting successful larval development [30,31]. However, when temperatures reached 34 °C, development time increased significantly and survival rates dropped sharply. We speculate that the occurrence of developmental delays and increased mortality in the larvae might be related to their prolonged exposure to high temperatures, consistent with observations in other insect species nearing their thermal limits [32,33]. The results of this study align with the general pattern observed in most insects, which exhibit developmental inhibition and reduced survival rates as they approach their thermal tolerance limits [34,35]. However, the developmental duration of *M. hieroglyphica* larval measured in this study was longer than that reported by Li et al. [36]. We speculated that conspecific populations from different geographical regions or different years may vary in their responses to high temperatures. Food consumption is an important indicator of an insect’s physiological condition and energy metabolism, providing insights into their ability to adapt to varying temperatures. Previous research on *Drosophila melanogaster* [37] and *Athetis lepigone* [38] has shown that larval food intake declines significantly outside their optimal temperature ranges. Similarly, in this study, food consumption decreased significantly when temperatures exceeded 28 °C, indicating that the range of 25–28 °C is optimal for the development of *M. hieroglyphica*.

Elevated temperatures have a significant impact on the successful metamorphosis of insect larvae, leading to a decrease in the overall population reproduction rate. This negative effect is observed throughout the entire process of larval pupation and pupal emergence, making it a crucial factor in population growth [39]. Our research has revealed that when kept at an optimal temperature of 28 °C, the rates of pupation and emergence for *M. hieroglyphica* larvae remain consistently high, above 85%. Their physiological metabolism remains stable at this temperature, allowing them to complete critical developmental stages such as molting, pupation, and emergence successfully [40]. However, as temperatures rise to 31 °C and beyond, the rates of pupation and emergence begin to decline significantly. In experimental groups exposed to 34 °C, we observed instances of failed pupation, abnormalities in pupal morphology, decreased adult vitality, and premature mortality. Based on the above research results, we speculate that high temperatures not only directly affect the survival of larvae, but also have a lasting and irreversible impact on the development of pupae. Prolonged exposure to such stressors can ultimately lead to decreased reproductive capacity and effective population size, disrupting the delicate balance of ecosystems [34,41,42].

Continuous exposure to high temperatures can disrupt the delicate balance of an insect’s endocrine system, leading to the inhibition of normal hormone secretion in the brain. This disruption affects the regulation of crucial hormones involved in molting and development, such as brain hormones, molting hormones, and juvenile hormones. Consequently, the insect’s molting rhythm is disturbed, leading to incomplete pupal body development and ultimately reducing the success rate of pupation and emergence [43]. Furthermore, high temperatures can trigger excessive accumulation of reactive oxygen species in insects, disrupting cellular homeostasis and damaging the cellular structures of larvae and pupae. This oxidative damage subsequently interferes with the normal expression of genes related to metamorphosis, further hindering the developmental process from the perspective of oxidative stress [44]. Previous studies have shown that temperatures of 35 °C and above can significantly impede the pupation and emergence of *Agrotis ipsilon* [45], while acute temperatures of 38 °C can decrease the survival rate of bee pupae [46]. High temperatures have also been found to directly inhibit the activity of hormones related to pupation and prolong the pupal stage of soybean aphids, thereby reducing their emergence rate [47,48]. The findings of these studies align closely with the results of our research, collectively confirming that high temperatures have a widespread and substantial inhibitory effect on insect metamorphic development through both endocrine disruption and oxidative damage mechanisms. The body weight and length of adult insects serve as critical indicators for assessing the quality of insect growth and development, directly impacting their reproductive capacity and overall population fitness. Atkinson’s “Temperature-Size Rule” suggests that in ectothermic animals, development under higher temperatures often leads to smaller adult body sizes [49]. This rule was further corroborated in our study, providing additional evidence of the relationship between temperature and insect development.

In response to high-temperature stress, larvae of *M. hieroglyphica* display distinct patterns of antioxidant stress response. In the temperature range of 25–28 °C, the activities of four key antioxidant enzymes—catalase (CAT), superoxide dismutase (SOD), peroxidase (POD), and glutathione S-transferase (GST)—remain stable. This suggests that the larvae are able to effectively maintain the balance of reactive molecules within their cells at this range, preventing oxidative stress from occurring [50]. However, as temperatures rise to 31–34 °C, the activities of SOD and CAT show significant increases. This indicates that the higher temperatures lead to an accumulation of reactive oxygen species (ROS), prompting the activation of the primary antioxidant defense mechanism centered around SOD-CAT, aimed at neutralizing superoxide anions and hydrogen peroxide [41,51,52]. Interestingly, the activity of POD initially increases before decreasing, while GST activity continues to decline. We speculate that high-temperature stress can have different effects on various antioxidant and detoxification enzyme systems. Moderate high temperatures may stimulate POD to participate in secondary antioxidant reactions, while extreme temperatures could hinder enzyme protein synthesis, accelerate enzyme denaturation, or deplete cofactors, leading to reduced POD activity and weakened detoxification and oxidative damage repair functions mediated by GST [53,54]. This intricate pattern of changes in enzyme activity not only highlights the adaptability and tolerance limits of *M. hieroglyphica* larvae under high-temperature conditions but also lays the groundwork for delving deeper into the molecular and biochemical mechanisms behind their ability to thrive in such environments [55,56].

## 5. Conclusions

This study investigated the effects of different temperatures (25, 28, 31, and 34 °C) on the survival rate, food consumption (third instar), pupation rate, emergence rate, weight, body length, and antioxidant capacity of *M. hieroglyphica* larvae at various developmental stages. The results showed that high temperatures (31 °C and 34 °C) negatively affected developmental duration, survival rates, and food consumption in third-instar leaf beetle larvae across all stages. Temperature regulation effects were also evident in pupation rate, emergence rate, weight, and body length: the highest values for these parameters occurred at 28 °C, with a continuous decline as temperature increased. These changes were further reflected in the larvae’s antioxidant capacity, with the activities of SOD, CAT, GST, and POD being modulated by temperature. This study identified the optimal temperature range and the threshold for high-temperature stress in *M. hieroglyphica* larvae, elucidated their physiological and ecological response patterns, and provided key biological parameters for predicting population dynamics, occurrence periods, and outbreak risks of this pest under climate warming. Additionally, it offers a scientific basis for developing precise monitoring, early warning, and sustainable management strategies based on temperature regulation.

## Figures and Tables

**Table 1:** Effects of different temperatures on the developmental duration and mortality of *Monolepta hieroglyphica* (*M. hieroglyphica)* larvae.

	First		Second		Third	
	Developmental Duration (d)	Mortality (%)	Developmental Duration (d)	Mortality (%)	Developmental Duration (d)	Mortality (%)
25 °C	9.59 ± 0.33 a	5.66 ± 0.16 c	8.82 ± 0.33 a	13.06 ± 0.39 bc	10.38 ± 0.70 ab	30.77 ± 3.81 b
28 °C	8.29 ± 0.31 b	4.92 ± 0.11 c	7.63 ± 0.10 b	7.02 ± 2.48 c	9.90 ± 0.29 b	8.17 ± 3.06 c
31 °C	7.59 ± 0.12 b	16.08 ± 0.41 b	6.26 ± 0.07 c	18.10 ± 2.69 b	9.44 ± 0.33 b	25.67 ± 0.94 b
34 °C	7.89 ± 0.21 b	32.01 ± 1.85 a	7.49 ± 0.51 b	28.33 ± 3.40 a	11.75 ± 0.25 a	66.36 ± 6.36 a

Note: The results are represented as mean ± SE. Means followed by the same letter within the same column are not significantly different (ANOVA: Tukey’s post hoc test, *p* < 0.05) between treatments.

## Data Availability

All data analyzed in this study are included in this article.

## Supplementary Materials

The following supporting information can be downloaded at: https://www.mdpi.com/article/10.3390/insects17050489/s1, Figure S1: Standard curve of antioxidant enzyme activity levels; Table S1: Effects of temperature stress on the detoxification enzyme activity levels of larvae *Monolepta hieroglyphica* at different instars.
