# Supplementary material for: Effects of High Temperature on Development, Survival, and Antioxidant Responses of Immature Monolepta hieroglyphica
Source: Insects. 2026 May 11;17(5):489. doi: 10.3390/insects17050489 (PMC13207502; doi:10.3390/insects17050489)
Supplement: Supplementary file 1 [file insects-17-00489-s001.zip › insects-4256005-supplementary.pdf]

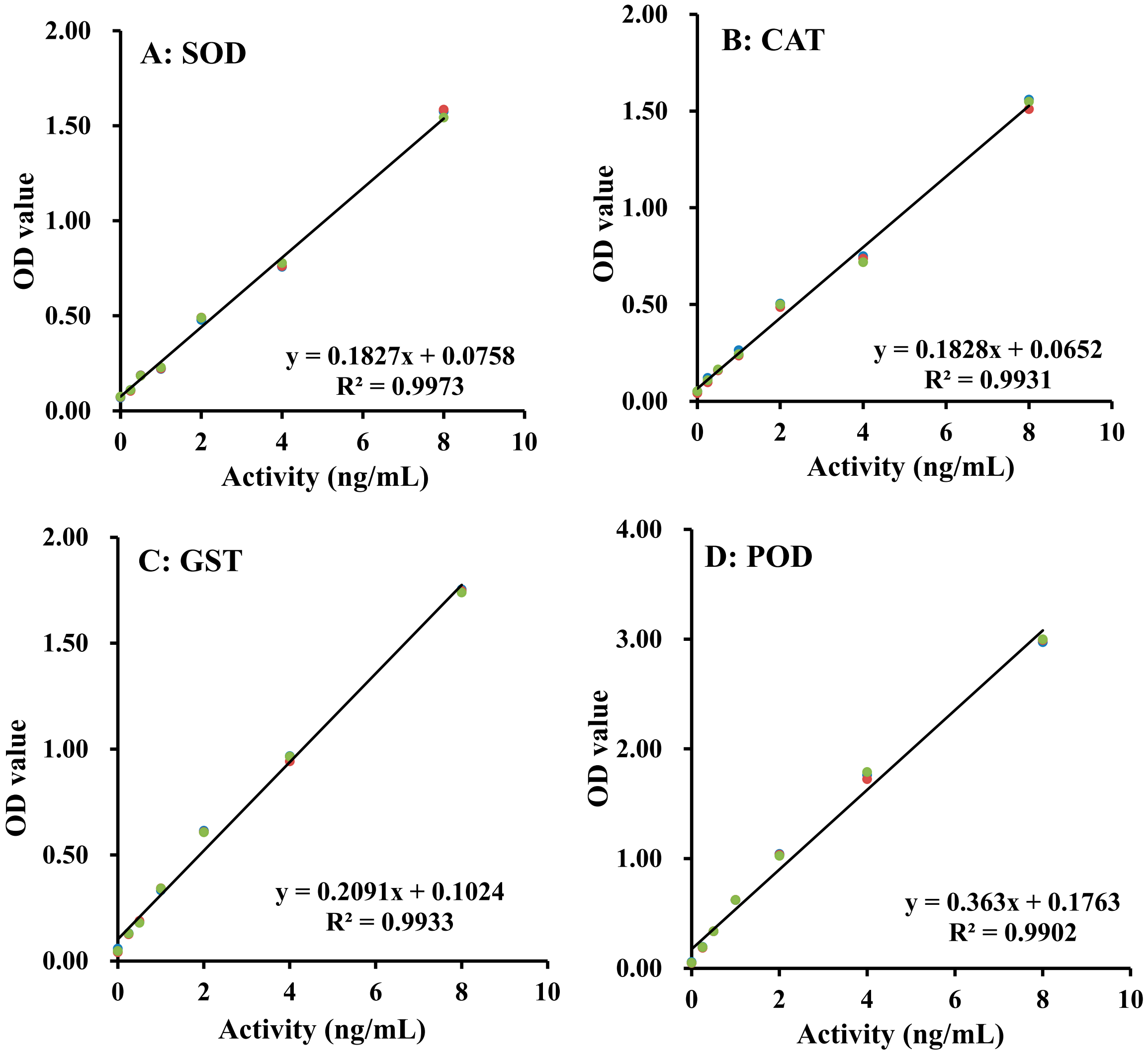

Supplemental Figure S1: Standard curve of antioxidant enzyme activity levels

Supplemental Table S1: Effects of temperature stress on the detoxification enzyme activity levels of larvae *Monolepta hieroglyphica* at different instars.

		Temperature			
		25°C	28°C	31°C	34°C
CAT	1age	9.25±0.40ab	9.15±0.02a	10.99±0.27bc	11.77±0.16c
	2age	9.74±0.32a	9.87±0.05ab	10.46±0.22b	11.64±0.29c
	3age	9.19±0.17a	9.09±0.15a	10.02±0.13b	12.76±0.08c
GST	1age	8.76±0.05c	8.66±0.04c	7.94±0.08b	7.00±0.03a
	2age	8.54±0.03c	8.59±0.19c	7.41±0.15b	6.97±0.23a
	3age	9.15±0.05c	9.00±0.09c	8.36±0.16b	4.91±0.13a
SOD	1age	8.79±0.20ab	8.56±0.18a	9.17±0.16c	9.91±0.12d
	2age	8.69±0.15a	8.67±0.10a	8.62±0.07a	11.89±0.20b
	3age	8.37±0.18a	8.03±0.14a	9.73±0.15b	11.03±0.02c
POD	1age	2.25±0.13b	2.25±0.07b	3.94±0.13c	1.91±0.04a
	2age	2.28±0.12b	2.19±0.07b	3.29±0.12c	1.60±0.04a
	3age	2.24±0.06b	2.18±0.10b	3.30±0.08c	1.62±0.04a

Note: means followed by the same letter within the same column are not significantly different (ANOVA: Tukey's post hoc test, $P < 0.05$) between treatments.
